# A Kind of (*t*, *n*) Threshold Quantum Secret Sharing with Identity Authentication

**DOI:** 10.3390/e25050827

**Published:** 2023-05-22

**Authors:** Depeng Meng, Zhihui Li, Shuangshuang Luo, Zhaowei Han

**Affiliations:** School of Mathematics and Statistics, Shaanxi Normal University, Xi’an 710119, China; mdp@snnu.edu.cn (D.M.);

**Keywords:** quantum secret sharing, identity authentication, mutually unbiased bases, (*t*, *n*) threshold scheme

## Abstract

Quantum secret sharing (QSS) is an important branch of quantum cryptography. Identity authentication is a significant means to achieve information protection, which can effectively confirm the identity information of both communication parties. Due to the importance of information security, more and more communications require identity authentication. We propose a *d*-level (t,n) threshold QSS scheme in which both sides of the communication use mutually unbiased bases for mutual identity authentication. In the secret recovery phase, the sharing of secrets that only the participant holds will not be disclosed or transmitted. Therefore, external eavesdroppers will not get any information about secrets at this phase. This protocol is more secure, effective, and practical. Security analysis shows that this scheme can effectively resist intercept–resend attacks, entangle–measure attacks, collusion attacks, and forgery attacks.

## 1. Introduction

Secret sharing is an important research field in cryptography. It has important applications in many aspects, such as network communication, signature checking, and identity verification. In 1979, Shamir [1] proposed the first secret-sharing protocol based on Lagrange interpolation formula. With the rapid development of quantum technology, quantum secret sharing (QSS) has also made great progress. In 1999, Hillery et al. [2] proposed the first QSS protocol using the Greenberger–Horne–Zeilinger (GHZ) state. Since then, more and more relatively complete QSS protocols [3,4,5,6,7,8,9,10,11,12,13,14,15,16,17] have been proposed by scholars. Like the (n,n) threshold QSS protocol [3,4,5], the secret is divided into *n* parts. Only *n* participants can cooperate to recover the secret. However, due to practical needs and consideration of flexibility, some (t,n) threshold QSS protocols [6,7,8,9,10,11,12,13,14,15,16,17] have received great attention. The secret is also divided into *n* parts, but *t* participants can recover the secret and fewer than *t* participants cannot recover the secret. In addition, to detect the existence of external attackers and check the integrity of internal participants, some verifiable QSS protocols [11,12,13,14,15,16,17] have been proposed. They mainly include message authentication (verify the correctness of the message) and identity authentication (verify the correctness of identity). Identity authentication is a systematic process to verify the identity of legitimate users, components and devices. Therefore, it is the security guarantee of various encryption tasks. In the identity authentication scheme, the sender registers the secret information as his identity information in the receiver’s database before communication. Afterwards, the sender proves the secret identification information to the receiver, that is, his identity information. The receiver can prove that the sender is a legitimate user before establishing the communication channel by using an authentication scheme, so he avoids the occurrence of an illegal sender. In quantum cryptography, quantum secret sharing [15,16,17], quantum key distribution [18,19,20,21], quantum secure direct communication [22,23], etc., all require identity authentication. In real life, the importance of identity authentication is also reflected everywhere.

In 2013, Yang et al. [3] constructed an QSS using entangled state and quantum Fourier transform (QFT). In 2015, Tavakoli [4] proposed a *d*-level QSS based on GHZ state and mutually unbiased bases. The above two schemes are (n,n) threshold. In 2017, Song et al. [7] proposed a *d*-level (t,n) threshold QSS based on Shamir’s secret-sharing scheme and the Lagrange interpolation formula. However, restricted by private secret shares, the scheme is infeasible. In 2020, Sutradhar et al. [8] proposed an QSS without credible participants. Nevertheless, in the actual process, the reconstructor needs to compare secrets and the hash value of secrets, so the reconstructor must be trustworthy. In 2020, Mashhadi [9] pointed out the problems in the protocol of Song et al. [7] and gave an improvement scheme. In this improved protocol, each participant applies the inverse quantum Fourier transform (IQFT) on its own particle. Then, each participant measures and publishes the measurement results. At this time, everyone can recover the original secret, but there is no identity authentication process in the transmission of quantum states, and we cannot guarantee that the corresponding operation is performed by the corresponding participant. In 2021, Hu et al. [17] proposed a dynamic QSS using GHZ state in a high-dimensional quantum system. In this protocol, each participant performs corresponding unitary operations according to its own measurement results.

In this paper, we overcome the above problems. The innovation of this article is to improve [8] by combining relevant knowledge. We mainly add identity authentication content to make the protocol more secure and complete. Our protocol is a *d*-level (t,n) threshold scheme that both parties can be mutually verified. Each participant can act as a reconstructor to recover the secret. When a participant wants to recover the secret, he can cooperate with participants in an authorized subset to obtain the secret. The direct communication parties will conduct mutual identity authentication through mutually unbiased bases. After passing the authentication, other participants use direct product operation on their own particles and auxiliary particle passed by the reconstructor. Then, the reconstructor measures the final secret after performing the IQFT. Finally, he verifies whether the correct secret is obtained by comparing the secret and the hash value of the secret published by the dealer.

The rest of the article is organized as follows. In Section 2, we give the preliminary knowledge needed for this article. In Section 3, we propose a (t,n) threshold quantum secret sharing scheme with identity authentication. In Section 4, we give the correctness proof of the agreement. In Section 5, we analyze the security of the protocol. In Section 6, we compare and analyze this protocol with some previous protocols. In Section 7, we give a specific example to better understand the protocol. In Section 8, we summarize the full text and draw conclusions.

## 2. Preliminaries

In this section, we introduce some basic knowledge needed in this article, including quantum measurement, mutually unbiased bases, QFT, IQFT, and CNOT operation.

### 2.1. Quantum Measurement

Quantum measurement can be described based on a set of measurement operators $\left\{M_{i}\right\}$. These measurement operators satisfy the completeness equation:

(1) $$\sum_{i}M_{i}^{†}M_{i}=1.$$

When the quantum state $|\varphi \rangle$ is measured, the probability that the result is *i* is:

(2) $$p\left(m\right)=\langle \varphi |M_{i}^{†}M_{i}|\varphi \rangle .$$

After measurement, the quantum state collapses as follows:

(3) $${|\varphi \rangle }^{\prime }=\frac{M_{i}|\varphi \rangle }{\sqrt{\langle \varphi |M_{i}^{†}M_{i}|\varphi \rangle }}.$$

Therefore, quantum measurement will change the original state of the quantum state.

### 2.2. Mutually Unbiased Bases

Let *d* be an odd prime number and $Z_{d}$ be a finite field. Suppose $V_{1}=\{|u_{i}\rangle {\}}_{i=1}^{d}$, $V_{2}=\{|v_{j}\rangle {\}}_{j=1}^{d}$ are two sets of standard orthogonal bases on *d*-dimensional Hilbert space. If they satisfy:

(4) $$|\langle u_{i}|v_{j}\rangle |=\frac{1}{\sqrt{d}}.$$

Then these two groups of bases are called mutually unbiased bases. If any two sets of bases in $V=\{V_{1},V_{2},\cdots ,V_{m}\}$ are mutually unbiased, *V* is called mutual unbiased bases set. Additionally, there are at most d+1 elements in set *V*. Specifically, the calculation base {|z〉}, $z\in Z_{d}$, is one of them. The remaining *d* groups can be expressed as:

(5) $$|e_{l}^{j}\rangle =\frac{1}{\sqrt{d}}\sum_{z=0}^{d-1}\omega^{z(l+jz)}|z\rangle ,$$

where l,j∈{0,1,⋯,d−1}, $\omega =e^{\frac{2\pi i}{d}}$, *j* represents the sequence of bases, and *l* represents vector sequence in a set of bases. They satisfy the following relation:

(6) $$|\langle e_{l}^{j}|e_{l^{\prime }}^{j^{\prime }}\rangle |=\frac{1}{\sqrt{d}},j\neq j^{\prime }.$$

Additionally, among mutually unbiased bases, the following unitary operation makes them transform each other:

(7) $$X_{d}=\sum_{u=0}^{d-1}\omega^{u}|u\rangle ,Y_{d}=\sum_{u=0}^{d-1}\omega^{u^{2}}|u\rangle ,$$

let

(8) $$U_{x,y}=X_{d}^{x}Y_{d}^{y}.$$

We have

(9) $$U_{x,y}|e_{l}^{j}\rangle =|e_{l+x}^{j+y}\rangle .$$

### 2.3. QFT, IQFT

The QFT in the *d*-dimensional system can be expressed as follows:

(10) $$F|x\rangle =\frac{1}{\sqrt{d}}\sum_{y=0}^{d-1}\omega^{x\cdot y}|y\rangle .$$

where $\omega =e^{\frac{2\pi i}{d}}$, $x,y\in Z_{d}$. Similarly, the IQFT can be expressed as:

(11) $$F^{-1}|x\rangle =\frac{1}{\sqrt{d}}\sum_{y=0}^{d-1}\omega^{-x\cdot y}|y\rangle .$$

It is easy to know that both discrete QFT and discrete IQFT are unitary transformations. In addition, by

(12) $$\begin{array}{c} \sum_{q=0}^{d-1}\omega^{sq}=\left\{\begin{array}{cc} 0, & s\neq 0\;\mathrm{mod}\;d,\\ d, & s=0\;\mathrm{mod}\;d, \end{array}\right. \end{array}$$

We can obtain

(13) $$F^{-1}(F|x\rangle )=|x\rangle .$$

### 2.4. CNOT Operation

CNOT is a two-qubit gate. In the *d*-dimensional system, it can be expressed as follows:

(14) $$CNOT(|x_{1}\rangle ,|x_{2}\rangle )=(|x_{1}\rangle ,|x_{1}⊕x_{2}\rangle ),$$

where $|x_{1}\rangle$ is control bit, $|x_{2}\rangle$ is the target bit, $x_{1},x_{2}\in Z_{d}$.

## 3. Proposed Protocol

**Figure 1:**
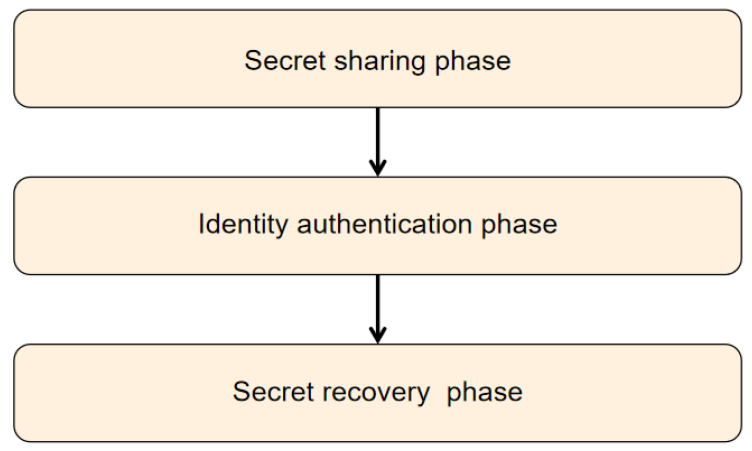
The process of this scheme.

In this section, we propose a quantum secret-sharing scheme with *d*-level and (t,n) threshold. Participants can verify each other mutually. Dealer Alice distributes secret shares among the set of participants B = {Bob${}_{1}$,Bob${}_{2}$,⋯,Bob${}_{n}$}. At least *t* participants can recover the secret. As the participants mutually verify, the protocol is more secure and practical. The entire scheme consists of three stages, namely the secret-sharing stage, identity authentication stage, and secret-recovery stage. The continuous identity authentication is included in the entire secret-recovery phase. Here, we use Figure 1 to briefly represent the entire process. The specific scheme of the protocol is shown below.

### 3.1. Secret-Sharing Phase

In this phase, The dealer Alice performs the following operations:

**(I)** Alice selects a binary symmetric polynomial F(x,y) of degree (t−1) in the $Z_{d}$. The (t−1) degree polynomial can be defined as:

(15) $$F\left(x,y\right)=S+a_{10}x+a_{01}y+a_{20}x^{2}+a_{02}y^{2}+a_{11}xy+\cdots +a_{t-1,t-1}x^{t-1}y^{t-1},$$

where $Z_{d}$ is a finite field, *S* is secret, *d* is an odd prime number, coefficients $a_{ij}\in Z_{d}$, $a_{ij}=a_{ji}$, i,j∈{0,1,⋯,t−1}.

**(II)** Alice calculates polynomials F($x_{i}$,y) (i=1,2,⋯,n), respectively, by (15) and sends them to the corresponding participants Bob${}_{i}$ through a secure classical channel, where $x_{i}\in Z_{d}$ is the public identity information of the corresponding participant Bob${}_{i}$ with $x_{i}\neq x_{j}$ for i≠j.

**(III)** According to the characteristics of binary symmetric polynomials, we define the following two groups of constants:

(16) $$k_{i,j}=F\left(x_{i},x_{j}\right)=F\left(x_{j},x_{i}\right)=k_{j,i},$$

(17) $$sk_{i,j}=F\left(x_{i},x_{j}\right)=F\left(x_{j},x_{i}\right)=vk_{j,i}.$$

**Remark** **1.** *Here, these four values are the same. However, in the following text, different symbols have different meanings. $k_{i,j}$ and $k_{j,i}$ represent the symmetry keys during encryption and decryption. $sk_{i,j}$ and $vk_{j,i}$ represent one’s own identity information, used to indicate one’s identity, which can be understood as one’s own signature information.*

**(IV)** Alice chooses a one-way hash function h(). Then, Alice discloses the hash algorithm and hash value H=h(S) of the secret *S*.

### 3.2. Secret-Recovery Phase

Suppose Bob${}_{1}$(reconstructor) wants to get the secret *S*. Then at least another t−1 participants need to be selected to form a qualified subset with him to jointly recover the secret S. Let us suppose B${}_{1}$ = {Bob${}_{1}$, Bob${}_{2}$, ⋯, Bob${}_{t}$} is a qualified subset from all the qualified subsets. Each participant in the set has the ability to independently produce a single photon. The corresponding participant will perform the following processes to recover the secret:

**(I)** Each participant Bob${}_{i}$, i=(1,2,⋯,t), calculates the shadow $\left(S_{i}\right)$ of the share according to own polynomial and prepares computational basis state $|S_{i}\rangle$ with *d*-level.

(18) $$S_{i}=F\left(x_{i},0\right)\prod_{j\neq i}^{t}\frac{x_{j}}{x_{j}-x_{i}}\;\mathrm{mod}\;d.$$

**Remark** **2.** *Here, $\frac{1}{x_{j}-x_{i}}$ is the modular multiplicative inverse of the integer $\left(x_{j}-x_{i}\right)$. According to the recent literature, this calculation has a fast calculation method. We will not expand here as readers can refer to [24].*

**(II)** Bob${}_{1}$ applies QFT on the computational basis state $|S_{1}\rangle$ and gets the result $|\phi_{1}\rangle$.

(19) $$|\phi_{1}\rangle =\mathrm{QFT}(|S_{1}\rangle )=\frac{1}{\sqrt{d}}\sum_{k=0}^{d-1}\omega^{S_{1}k}|k\rangle .$$

**(III)** Bob${}_{1}$ again prepares computational basis state |0〉 with *d*-level and performs CNOT operation according to $|\phi_{1}\rangle$ and |0〉. $|\phi_{1}\rangle$ is the control bit and |0〉 is the target bit. When the operation is completed, Bob${}_{1}$ obtains the entangled state $|\phi_{2}\rangle$.

(20) $$|\phi_{2}\rangle =CNOT(|\phi_{1}\rangle ,|0\rangle )=CNOT(\frac{1}{\sqrt{d}}\sum_{k=0}^{d-1}\omega^{S_{1}k}|k\rangle ,|0\rangle )=\frac{1}{\sqrt{d}}\sum_{k=0}^{d-1}\omega^{S_{1}k}{|k\rangle }_{H}{|k\rangle }_{T}.$$

The subscript *H* and *T* here are used to distinguish two particles.

**(IV)** Bob${}_{1}$ and Bob${}_{2}$ mutually conduct identity authentication:

**Step 1.** Bob${}_{1}$ prepares a *d*-level initial quantum state $|e_{0}^{0}\rangle$, two random numbers $c_{1}$, $p_{1}$, and opens $p_{1}$. Bob${}_{1}$ performs the unitary transformation $U_{p_{1},c_{1}}$ on the initial quantum state and obtains a new quantum state $|\Psi_{1}\rangle =U_{p_{1},c_{1}}|e_{0}^{0}\rangle =|e_{p_{1}}^{c_{1}}\rangle$. Then according to own polynomial $F(x_{1},y)$, Bob${}_{1}$ can obtain $sk_{1,2}=F\left(x_{1},x_{2}\right)$. Subsequently, Bob${}_{1}$ performs the unitary transformation $U_{sk_{1,2},0}$ on $|\Psi_{1}\rangle$ and obtains $|\Psi_{1,2}\rangle =|e_{p_{1}+sk_{1,2}}^{c_{1}}\rangle$. Bob${}_{1}$ again determines a random moment $t_{1,2}$. Lastly, Bob${}_{1}$ sends messages $E_{k_{1,2}}\left(c_{1},t_{1,2}\right)$, which has been encrypted, and $|\Psi_{1,2}\rangle$ to Bob${}_{2}$ through secure classical channel and quantum channel, respectively.

**Step 2.** After Bob${}_{2}$ receives the quantum state and encrypted information, he first calculates $vk_{2,1}=F\left(x_{2},x_{1}\right)$ according to the own polynomial $F(x_{2},y)$. Afterwards Bob${}_{2}$ performs the unitary transformation $U_{-vk_{2,1},0}$ on $|\Psi_{1,2}\rangle$ and obtains $|\Psi_{1}\rangle^{\prime }=|e_{p_{1}+sk_{1,2}-vk_{2,1}}^{c_{1}}\rangle$. Then, Bob${}_{2}$ obtains a number pair $\left(c_{1},t_{1,2}\right)=D_{k_{2,1}}\left(E_{k_{1,2}}\left(c_{1},t_{1,2}\right)\right)$ by decrypting the received classic information. Finally, Bob${}_{2}$ uses the basis $\{|e_{l}^{c_{1}}\rangle \}\left(l\in Z_{d}\right)$ to measure $|\Psi_{1}\rangle^{\prime }$ to obtain the measurement result ${\left(p_{1}\right)}^{\prime }$ and compares ${\left(p_{1}\right)}^{\prime }$ with the published random number $p_{1}$. If ${\left(p_{1}\right)}^{\prime }=p_{1}$; then, Bob${}_{2}$ considers that all the information comes from Bob${}_{1}$. The identity information of Bob${}_{1}$ is authenticated. Otherwise, Bob${}_{2}$ considers that the message does not come from Bob${}_{1}$ or is destroyed in the middle of the process and terminates this agreement.

**Step 3.** After Bob${}_{2}$ confirms that the message originated from Bob${}_{1}$, he also prepares a *d*-level initial quantum state $|e_{0}^{0}\rangle$, two random numbers $c_{2}$, $p_{2}$, and opens $p_{2}$. Then, Bob${}_{2}$ performs the unitary transformation $U_{p_{2},c_{2}}$ on $|e_{0}^{0}\rangle$ and obtains a new quantum state $|\Psi_{2,1}\rangle =U_{p_{2},c_{2}}|e_{0}^{0}\rangle =|e_{p_{2}}^{c_{2}}\rangle$. Bob${}_{2}$ decides another moment $t_{2,1}$ and sends encrypted message $E_{k_{2,1}}\left(c_{2},t_{2,1}\right)$ to Bob${}_{1}$. Lastly, Bob${}_{2}$ is ready to send $|\Psi_{2,1}\rangle$ to Bob${}_{1}$ at moment $t_{2,1}$.

**Figure 2:**
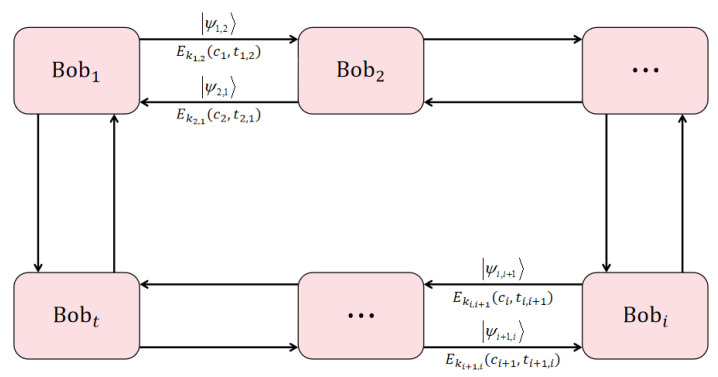
Identity authentication process between participants in this scheme.

**Step 4.** Bob${}_{1}$ decrypts the encrypted classical information to obtain a random number pair $\left(c_{2},t_{2,1}\right)=D_{k_{1,2}}\left(E_{k_{2,1}}\left(c_{2},t_{2,1}\right)\right)$. After receiving the message particle from Bob${}_{2}$ at moment $t_{2,1}$, Bob${}_{1}$ selects the basis $\{|e_{l}^{c_{2}}\rangle \}\left(l\in Z_{d}\right)$ to measure $|\Psi_{2,1}\rangle$ to obtain the measurement result ${\left(p_{2}\right)}^{\prime }$ and compares ${\left(p_{2}\right)}^{\prime }$ with the published random number $p_{2}$. If ${\left(p_{2}\right)}^{\prime }=p_{2}$, Bob${}_{1}$ believes that all the information comes from Bob${}_{2}$ and Bob${}_{2}$ has received an own message. So, Bob${}_{1}$ will send the auxiliary state ${|k\rangle }_{T}$ in his own hand to Bob${}_{2}$ through the secure quantum channel at moment $t_{1,2}$. The entire identity authentication process is shown in Figure 2 below:

**Remark** **3.** *Here, secure quantum channel refers to a quantum channel that is not subject to external interference. That is, an authenticated quantum channel. Participants can engage in quantum direct communication.*

**(V)** After Bob${}_{2}$ receives ${|k\rangle }_{T}$ at moment $t_{1,2}$, he treats ${|k\rangle }_{T}$ as the control bit and $|S_{2}\rangle$ as the target bit. Then, Bob${}_{2}$ performs controlled black box operation $C_{k}$ on these two quantum states, where $C_{k}$ can be expressed as:

(21) $$C_{k}{:|k\rangle }_{T}|S_{2}{\rangle \to |k\rangle }_{T}U^{k}|S_{2}\rangle .$$

*U* is a linear transformation and it satisfies $U|S_{2}\rangle =\omega^{S_{2}}|S_{2}\rangle$. That is to say, $|S_{2}\rangle$ is an eigenvector of *U* with an eigenvalue of $\omega^{S_{2}}$. After performing the controlled black box operation, Bob${}_{2}$ next conducts the direct product operation of $|S_{2}\rangle$ and ${|k\rangle }_{T}$. Then, the whole quantum state system becomes $|\phi_{3}\rangle$.

(22) $$\begin{array}{cc} |\phi_{3}\rangle & =\left(I⊗I⊗C_{k}\right)(\frac{1}{\sqrt{d}}\sum_{k=0}^{d-1}\omega^{S_{1}k}|k\rangle_{H}{|k\rangle }_{T}|S_{2}\rangle )\\ \; & =\frac{1}{\sqrt{d}}\sum_{k=0}^{d-1}\omega^{S_{1}k}{|k\rangle }_{H}{|k\rangle }_{T}U^{k}|S_{2}\rangle \\ \; & =\frac{1}{\sqrt{d}}\sum_{k=0}^{d-1}\omega^{S_{1}k}{|k\rangle }_{H}{|k\rangle }_{T}\omega^{S_{2}k}|S_{2}\rangle \\ \; & =\frac{1}{\sqrt{d}}\sum_{k=0}^{d-1}\omega^{(S_{1}+S_{2})k}{|k\rangle }_{H}{|k\rangle }_{T}|S_{2}\rangle . \end{array}$$

**(VI)** Each participant, Bob${}_{i}$ and Bob${}_{i+1}$, repeat the above mutual authentication and operation process of Bob${}_{1}$ and Bob${}_{2}$. When Bob${}_{2}$ and Bob${}_{3}$ complete mutual authentication, Bob${}_{2}$ will send the auxiliary state ${|k\rangle }_{T}$ in his own hand to Bob${}_{3}$ through the secure quantum channel at moment $t_{2,3}$. Bob${}_{3}$ also performs a similar controlled black box operation first. Then, he performs the direct product operation on his quantum state $|S_{3}\rangle$ and the whole quantum system, and so on, until the last participant Bob${}_{t}$ completes the direct product operation. At this time, the whole quantum system becomes $|\phi_{4}\rangle$.

(23) $$|\phi_{4}\rangle =\frac{1}{\sqrt{d}}\sum_{k=0}^{d-1}\omega^{(\sum_{i=1}^{t}S_{i})k}{|k\rangle }_{H}{|k\rangle }_{T}|S_{2}\rangle |S_{3}\rangle \cdots |S_{t}\rangle .$$

**(VII)** When Bob${}_{t}$ completes the direct product operation, Bob${}_{t}$ completes the identity authentication process with Bob${}_{1}$ in the same way. After completing the authentication operation, Bob${}_{t}$ retransmits the auxiliary state ${|k\rangle }_{T}$ back to Bob${}_{1}$ through a secure quantum channel. After Bob${}_{1}$ receives the auxiliary state ${|k\rangle }_{T}$ again, he performs CNOT operation on the two particles in his hand, where ${|k\rangle }_{H}$ is control bit and ${|k\rangle }_{T}$ is target bit. At this time, the whole quantum system becomes $|\phi_{5}\rangle$.

(24) $$\begin{array}{cc} |\phi_{5}\rangle & =(CNOT(\frac{1}{\sqrt{d}}\sum_{k=0}^{d-1}\omega^{(\sum_{i=1}^{t}S_{i})k}|k\rangle_{H}|k\rangle_{T}))|S_{2}\rangle |S_{3}\rangle \cdots |S_{t}\rangle \\ \; & =\frac{1}{\sqrt{d}}\sum_{k=0}^{d-1}\omega^{(\sum_{i=1}^{t}S_{i})k}{|k\rangle }_{H}{|0\rangle }_{T}|S_{2}\rangle |S_{3}\rangle \cdots |S_{t}\rangle . \end{array}$$

**(VIII)** Bob${}_{1}$ uses computational basis to measure the quantum state ${|k\rangle }_{T}$ which has been handled by the CNOT operation. If the measurement result is |0〉, Bob${}_{1}$ believes that his auxiliary particles have not been destroyed or replaced. Bob${}_{1}$ will continue to perform the following steps. Otherwise Bob${}_{1}$ has reason to believe that the auxiliary state is damaged or replaced during the transmission process, thus ending the entire agreement.

**(IX)** Bob${}_{1}$ applies IQFT on his first quantum state ${|k\rangle }_{H}$ and measures the output to obtain the final secret $S^{\prime }=\sum_{i=1}^{t}S_{i}\;\mathrm{mod}\;d$.

**(X)** Bob${}_{1}$ calculates $H^{\prime }=h\left(S^{\prime }\right)$ according to hash function h() released by Alice and compares it with public H=h(S). If $H^{\prime }=H$, $S^{\prime }$, the secret obtained by Bob${}_{1}$ is the real secret. If not, Bob${}_{1}$ has reason to believe that there is at least one dishonest participant, thus terminating the agreement.

## 4. Correctness Analysis

In this section, we show the correctness of the protocol in the secret recovery phase through two theorems.

**Theorem** **1.** *The sum of t shares of participants is the secret to be recovered.*

**Proof.**  According to the Lagrange interpolation formula, we have

(25) $$\begin{array}{cc} \sum_{i=1}^{t}S_{i}\;\mathrm{mod}\;d & =F\left(x_{1},0\right)\prod_{j=2}^{t}\frac{x_{j}}{x_{j}-x_{1}}+\cdots +F\left(x_{t},0\right)\prod_{j=1}^{t-1}\frac{x_{j}}{x_{j}-x_{t}}\;\mathrm{mod}\;d\\ \; & =F(0,0)\\ \; & =S. \end{array}$$

□

**Theorem** **2.** *When Bob${}_{1}$ applies the IQFT on the first quantum state ${|k\rangle }_{H}$ in his hand and measures the output result, he could gobtain the secret S.*

**Proof.** 
(26) $$\begin{array}{cc} \; & \mathrm{IQFT}⊗I(\frac{1}{\sqrt{d}}\sum_{k=0}^{d-1}\omega^{(\sum_{i=1}^{t}S_{i})k}|k\rangle_{H}{|0\rangle }_{T})\\ = & (\frac{1}{\sqrt{d}}\sum_{k=0}^{d-1}\omega^{(\sum_{i=1}^{t}S_{i})k}\mathrm{IQFT}|k\rangle_{H}{)|0\rangle }_{T}\\ = & (\frac{1}{\sqrt{d}}\sum_{k=0}^{d-1}\omega^{(\sum_{i=1}^{t}S_{i})k}\left(\frac{1}{\sqrt{d}}\sum_{l=0}^{d-1}\omega^{-lk}\right)|l\rangle_{H}{)|0\rangle }_{T}\\ = & \left(\frac{1}{d}\sum_{k=0}^{d-1}\sum_{l=0}^{d-1}\omega^{(\sum_{i=1}^{t}S_{i}-l)k}\right){|0\rangle }_{T}\\ = & (\frac{1}{d}\sum_{k=0}^{d-1}|\sum_{i=1}^{t}S_{i}\;\mathrm{mod}\;d\rangle_{H}+\frac{1}{d}\sum_{l=0,l\neq \sum_{i=1}^{t}S_{i}}^{d-1}\left(\sum_{k=0}^{d-1}\omega^{(\sum_{i=1}^{t}S_{i}-l)k}\right){|l\rangle }_{H}{)|0\rangle }_{T}\\ = & (|\sum_{i=1}^{t}S_{i}\;\mathrm{mod}\;d\rangle_{H}+\frac{1}{d}\sum_{l=0,l\neq \sum_{i=1}^{t}S_{i}}^{d-1}{0|l\rangle }_{H}{)|0\rangle }_{T}\\ = & |\sum_{i=1}^{t}S_{i}{\;\mathrm{mod}\;d\rangle }_{H}{|0\rangle }_{T}\\ = & {|F(0,0)\rangle }_{H}{|0\rangle }_{T}\\ = & {|S\rangle }_{H}{|0\rangle }_{T}. \end{array}$$

□

## 5. Security Analysis

In this section, we analyze the security of our scheme against quantum attacks [25,26,27,28,29].

### 5.1. Intercept–Resend Attack

Suppose that there is an eavesdropper, Eve, who wants to steal secret information by performing an intercept–resend attack. When Bob${}_{i}$ communicates with Bob${}_{i+1}$, there will be three quantum states interacting through the quantum channel. They are $|\Psi_{i,i+1}\rangle =|e_{p_{i}+sk_{i,i+1}}^{c_{i}}\rangle$, $|\Psi_{i+1,i}\rangle =|e_{p_{i+1}}^{c_{i+1}}\rangle$, and auxiliary state ${|k\rangle }_{T}$. When Eve intercepts $|\Psi_{i,i+1}\rangle$ and $|\Psi_{i+1,i}\rangle$, she needs to obtain information by measuring, but Eve does not know the measurement basis $c_{i}$ and $c_{i+1}$. If Eve arbitrarily chooses a set of bases to measure, the probability of success is $\frac{1}{d}$ when d→∞, $\frac{1}{d}\to 0$. Therefore, the possibility of success is negligible. Even if Eve succeeds, $|\Psi_{i,i+1}\rangle$ and $|\Psi_{i+1,i}\rangle$ are also just the quantum states needed for Bob${}_{i}$ and Bob${}_{i+1}$ to verify their identities. These two quantum states have no information about secrets. As for auxiliary state ${|k\rangle }_{T}$, it is only the control bit in the secret recovery process and also has no information about secrets. Therefore, the intercept–resend attack is not successful.

### 5.2. Entangle–Measure Attack

In this attack, the eavesdropper Eve prepares an auxiliary state |e〉. By using unitary transformation to entangle the auxiliary state |e〉 onto the transmission particle, Eve measures the auxiliary state and compares it with the original result to obtain relevant information about the secret. In our scheme, only particle ${|k\rangle }_{T}$ is transferred between participants in the secret recovery phase. Therefore, suppose that when Bob${}_{1}$ transfers particle ${|k\rangle }_{T}$ to Bob${}_{2}$, Eve performs the *d*-level CNOT operation to entangle the auxiliary state |e〉 to the particle ${|k\rangle }_{T}$. At this time, $|\phi_{2}\rangle$ becomes $|\phi_{2}\rangle^{\prime }$.

(27) $$|\phi_{2}\rangle^{\prime }=(CNOT{(|k\rangle }_{T},|e\rangle ))|\phi_{2}\rangle =\frac{1}{\sqrt{d}}\sum_{k=0}^{d-1}\omega^{S_{1}k}{|k\rangle }_{H}{|k\rangle }_{T}|k⊕e\rangle .$$

When Bob${}_{2}$ completes its own operation and transfers particle ${|k\rangle }_{T}$ to Bob${}_{3}$, Eve performs *d*-level CNOT operation again. Where particle ${|k\rangle }_{T}$ is the control bit and auxiliary state |k+e〉 is target bit. At this time, $|\phi_{3}\rangle$ becomes $|\phi_{3}\rangle^{\prime }$.

(28) $$\begin{array}{cc} |\phi_{3}\rangle^{\prime } & =(CNOT{(|k\rangle }_{T},|k⊕e\rangle ))|\phi_{3}\rangle \\ \; & =\frac{1}{\sqrt{d}}\sum_{k=0}^{d-1}\omega^{(S_{1}+S_{2})k}{|k\rangle }_{H}{|k\rangle }_{T}|S_{2}\rangle |k⊕k⊕e\rangle \\ \; & =\frac{1}{\sqrt{d}}\sum_{k=0}^{d-1}\omega^{(S_{1}+S_{2})k}{|k\rangle }_{H}{|k\rangle }_{T}|S_{2}\rangle |e\rangle . \end{array}$$

Next, Eve obtains the result *e* by measuring the auxiliary state particle. She concludes that the particles transmitted between participants are the same. The particle ${|k\rangle }_{T}$ has no information about sharing the secret. She cannot obtain any information about the secret. Therefore, the entangle–measure attack is not feasible.

### 5.3. Collusion Attack

In the collusion attack, some collusive participants want to obtain information about others’ sharing of secrets through cooperation. Then, they can obtain the original secret. In our protocol, the sharing of secrets is calculated by each participant Bob${}_{i}$ through the own share polynomial $F(x_{i},y)$. Each participant only knows his own share. In addition, the sharing of secrets will not be disclosed or transferred to other participants. As a consequence, it is impossible for participants to obtain the others’ sharing of secrets. So collusive attack is not feasible.

### 5.4. Forgery Attack

Suppose the participant Bob${}_{i}$ wants to perform a forgery attack. Then, in the identity authentication phase, to prove his identity to Bob${}_{i-1}$ and Bob${}_{i+1}$, Bob${}_{i}$ must use the correct authentication information. He cannot use forged information, or the agreement will end early. In the secret-recovery phase, on the one hand, if Bob${}_{i}$ forges an auxiliary state ${{|k\rangle }_{T}}^{\prime }$ and transmits it to Bob${}_{i+1}$, then the measurement result of Bob${}_{1}$ in **(VIII)** will not be |0〉. Bob${}_{1}$ believes that the auxiliary state has been damaged and terminates the agreement in advance. On the other hand, if Bob${}_{i}$ uses his sharing of $S_{i}$ to forge a false computational basis state ${|S_{i}\rangle }^{\prime }$, Bob${}_{1}$ will get the wrong secret $S^{\prime }$ eventually. By comparing $h(S^{\prime })\neq h(S)$, Bob${}_{1}$ believes that at least one participant is dishonest and ends the agreement. Therefore, our protocol can resist forgery attacks.

## 6. Scheme Comparison

In this section, we analyze the quantum resources needed by our protocol and compare it with some previous protocols.

The protocol of Yang et al. [3] operates in *d*-dimensional space; it is a (n,n) threshold scheme. The scheme needs (n−1) message particles and performs *n* number of QFT operations and *n* number of measure operations. It uses fewer quantum resources, but the scheme is not flexible enough. This scheme can resist any computational attack, but it cannot resist collusion attacks.

The protocol of Song et al. [7] operates in *d*-dimensional space, it is a (t,n) threshold scheme. The secret reconstructor prepares *t* message particles and distributes (t−1) number of them to the other participants. The reconstructor starts with an QFT. Until the other participants complete the operation, the reconstructor performs an IQFT and measures particles to obtain the secret. Finally, the reconstructor verifies it through the hash function. This protocol can resist various common attacks. However, after some calculation and analysis, due to the mutual entanglement between particles, simple IQFT cannot recover the secret.

The protocol of Sutradhar et al. [8] is *d* level with (t,n) threshold. Using the Lagrange interpolation formula, the reconstructor first applies QFT to a particle. After each participant adds its share to the whole recovery process, the reconstructor uses the IQFT to recover the secret and measures to obtain the secret. The whole secret recovery process is repeated twice using two polynomials to restore the secret and the hash value of the secret, respectively. Through this method, the reconstructor can verify the correctness of the message. However, the protocol must require a trusted reconstructor, so the protocol can not resist collusion attack and can resist other common attacks.

The protocol of Mashhadi et al. [9] is an improvement to the protocol of Song et al. [7]. The protocol points out the inadequacy of its entanglement and proposes an improved scheme. Since the IQFT performed by the reconstructor cannot obtain the secret, *t* participants are required to perform IQFT in the entanglement system and summarize the measurement results to obtain the initial secret. Therefore, the protocol cannot resist intercept–resend attacks and collusion attacks.

Our protocol is also *d* level with (t,n) threshold. The dealer uses the binary symmetric polynomial to distribute the share polynomial. Each participant can use its own share polynomial to calculate the secret share and complete the identity authentication process. The protocol uses 2t number of message particles to complete the mutual authentication process of both parties. Finally, the reconstructor restores the secret by performing IQFT and obtains the secret through measurement. Although our protocol uses more quantum resources, every step is necessary. The identity authentication process will make the protocol more secure and reliable. Our protocol can also resist some attacks well. The comparison of these protocols is shown in Table 1 below.

## 7. Example

In this section, in order to better understand our protocol, we give a quantum secret sharing scheme with (4,6) threshold. In this protocol, *t* = 4, *n* = 6, *d* = 17, *S* = 2.

### 7.1. Secret-Sharing Phase

Alice performs the following operations:

**(I)** Alice selects a binary symmetric polynomial F(x,y) of degree 3 in the $Z_{17}$.

(29) $$\begin{array}{cc} F(x,y)= & 2+7x+7y+3x^{2}+3y^{2}+9xy+4x^{3}+4y^{3}+5x^{2}y+5xy^{2}\\ \; & +10x^{3}y+10xy^{3}+8x^{2}y^{2}+3x^{3}y^{2}+3x^{2}y^{3}+15x^{3}y^{3}, \end{array}$$

where secret *S* = 2.

**(II)** Alice calculates polynomials F($x_{i}$,y) (i=1,2,⋯,6), respectively, by Equation (29) and sends them to the corresponding participants Bob${}_{i}$ through a secure channel, where $x_{i}=i$. Here, the polynomial obtained by each Bob${}_{i}$ is:

(30) $$\begin{array}{cc} \mathrm{Bob}{}_{1}:F(1,y) & =16+14y+2y^{2}+15y^{3};\\ \mathrm{Bob}{}_{2}:F(2,y) & =9+6y+y^{2}+3y^{3};\\ \mathrm{Bob}{}_{3}:F(3,y) & =5+9y+y^{2}+7y^{3};\\ \mathrm{Bob}{}_{4}:F(4,y) & =11+15y+3y^{2}+15y^{3};\\ \mathrm{Bob}{}_{5}:F(5,y) & =16y+8y^{2}+15y^{3};\\ \mathrm{Bob}{}_{6}:F(6,y) & =14+4y+12y^{3}. \end{array}$$

**(III)** According to the characteristics of binary symmetric polynomial, constants have the following relationship: $sk_{i,j}=vk_{j,i}=k_{i,j}=k_{j,i}=F\left(x_{i},x_{j}\right)=F\left(x_{j},x_{i}\right)$. According to the selected binary symmetric polynomial and the identity information of each participant, we can obtain:

(31) $$\begin{array}{cc} sk_{1,2}=vk_{2,1}=k_{1,2}=k_{2,1}=F\left(x_{1},x_{2}\right)=F\left(x_{2},x_{1}\right) & =2;\\ sk_{2,3}=vk_{3,2}=k_{2,3}=k_{3,2}=F\left(x_{2},x_{3}\right)=F\left(x_{3},x_{2}\right) & =15;\\ sk_{3,4}=vk_{4,3}=k_{3,4}=k_{4,3}=F\left(x_{3},x_{4}\right)=F\left(x_{4},x_{3}\right) & =12;\\ sk_{4,1}=vk_{1,4}=k_{4,1}=k_{1,4}=F\left(x_{4},x_{1}\right)=F\left(x_{1},x_{4}\right) & =10. \end{array}$$

**(IV)** Alice chooses a one-way hash function h(). Then, Alice discloses the hash algorithm and hash value H=h(2) of the secret *S* = 2.

### 7.2. Secret-Recovery Phase

Suppose Bob${}_{1}$(reconstructor) wants to get the secret *S*. Bob${}_{1}$ chooses Bob${}_{2}$, Bob${}_{3}$, and Bob${}_{4}$ to help him recover the secret. Each participant has the ability to independently produce a single photon.

**(I)** Each participant Bob${}_{i}$, i=(1,2,3,4), calculates the shadow $\left(S_{i}\right)$ of the share according to the own polynomial $F(x_{i},y)$.

(32) $$\mathrm{Bob}{}_{1}:S_{1}=F\left(1,0\right)\cdot \frac{2}{2-1}\cdot \frac{3}{3-1}\cdot \frac{4}{4-1}\;\mathrm{mod}\;17=13.$$

Similarly, $S_{2}$ = 14, $S_{3}$ = 3, $S_{4}$ = 6. Then, they separately prepare a 17-level computational basis state |13〉, |14〉, |3〉, and |6〉.

**(II)** Bob${}_{1}$ applies QFT on the computational basis state |13〉 and obtains the result $|\phi_{1}\rangle$.

(33) $$|\phi_{1}\rangle =\mathrm{QFT}(|13\rangle )=\frac{1}{\sqrt{17}}\sum_{k=0}^{16}\omega^{13k}|k\rangle .$$

**(III)** Bob${}_{1}$ again prepares computational basis state |0〉 with 17-levels and performs CNOT operation according to $|\phi_{1}\rangle$ and |0〉. $|\phi_{1}\rangle$ is the control bit and |0〉 is the target bit. When the operation is completed, Bob${}_{1}$ obtains the entangled state $|\phi_{2}\rangle$.

(34) $$|\phi_{2}\rangle =CNOT(|\phi_{1}\rangle ,|0\rangle )=CNOT(\frac{1}{\sqrt{17}}\sum_{k=0}^{16}\omega^{13k}|k\rangle ,|0\rangle )=\frac{1}{\sqrt{17}}\sum_{k=0}^{16}\omega^{13k}{|k\rangle }_{H}{|k\rangle }_{T}.$$

**(IV)** Bob${}_{1}$ and Bob${}_{2}$ mutually conduct identity authentication:

**Step 1.** Bob${}_{1}$ prepares a 17-level initial quantum state $|e_{0}^{0}\rangle$, 2 random numbers $c_{1}$ = 6, $p_{1}$ = 8, and opens $p_{1}$. Bob${}_{1}$ performs the unitary transformation $U_{p_{1},c_{1}}=U_{8,6}$ on the initial quantum state and obtains a new quantum state $|\Psi_{1}\rangle =U_{8,6}|e_{0}^{0}\rangle =|e_{8}^{6}\rangle$. Then, according to the own polynomial F(1,y), Bob${}_{1}$ can obtain $sk_{1,2}=F\left(1,2\right)=2$. Subsequently, Bob${}_{1}$ performs the unitary transformation $U_{2,0}$ on $|\Psi_{1}\rangle$ and obtains $|\Psi_{1,2}\rangle =U_{2,0}|e_{8}^{6}\rangle =|e_{10}^{6}\rangle$. Bob${}_{1}$ again determines a random moment $t_{1,2}=9$. Lastly, Bob${}_{1}$ sends message $E_{k_{1,2}}\left(6,9\right)$, which has been encrypted, and $|\Psi_{1,2}\rangle$ to Bob${}_{2}$ through secure classical channel and quantum channel, respectively.

**Step 2.** After Bob${}_{2}$ receives the quantum state and encrypted information, he first calculates $vk_{2,1}=F\left(2,1\right)=2$ according to the own polynomial F(2,y). Afterwards, Bob${}_{2}$ performs the unitary transformation $U_{-2,0}$ on $|\Psi_{1,2}\rangle$ and obtains $|\Psi_{1}\rangle^{\prime }=U_{-2,0}|e_{10}^{6}\rangle =|e_{10-2}^{6}\rangle =|e_{8}^{6}\rangle$. Then, Bob${}_{2}$ obtains a number pair $\left(6,9\right)=D_{k_{2,1}}\left(E_{k_{1,2}}\left(6,\right)\right)$ by decrypting the received classic information. Finally, Bob${}_{2}$ uses the basis $\{|e_{l}^{6}\rangle \}\left(l\in Z_{17}\right)$ to measure $|\Psi_{1}\rangle^{\prime }$ to obtain the measurement result ${\left(p_{1}\right)}^{\prime }$ and compares ${\left(p_{1}\right)}^{\prime }$ with the published random number $p_{1}=8$. If ${\left(p_{1}\right)}^{\prime }=8$, then Bob${}_{2}$ considers that all the information comes from Bob${}_{1}$. The identity information of Bob${}_{1}$ is authenticated. Otherwise, Bob${}_{2}$ considers that the message does not come from Bob${}_{1}$ or is destroyed in the middle of the process and terminates this agreement.

**Step 3.** After Bob${}_{2}$ confirms that the message originated from Bob${}_{1}$, he also prepares a 17-level initial quantum state $|e_{0}^{0}\rangle$, 2 random numbers $c_{2}=5$, $p_{2}=12$, and opens $p_{2}$. Then, Bob${}_{2}$ performs the unitary transformation $U_{p_{2},c_{2}}=U_{12,5}$ on $|e_{0}^{0}\rangle$ and obtains a new quantum state $|\Psi_{2,1}\rangle =U_{12,5}|e_{0}^{0}\rangle =|e_{12}^{5}\rangle$. Bob${}_{2}$ decides another moment $t_{2,1}=7$ and sends encrypted message $E_{k_{2,1}}\left(5,7\right)$ to Bob${}_{1}$. Lastly, Bob${}_{2}$ is ready to send $|\Psi_{2,1}\rangle$ to Bob${}_{1}$ at moment $t_{2,1}=7$.

**Step 4.** Bob${}_{1}$ decrypts the encrypted classical information to obtain a random number pair $\left(5,7\right)=D_{k_{1,2}}\left(E_{k_{2,1}}\left(5,7\right)\right)$. After receiving the message particle from Bob${}_{2}$ at moment $t_{2,1}=7$, Bob${}_{1}$ selects the basis $\{|e_{l}^{5}\rangle \}\left(l\in Z_{17}\right)$ to measure $|\Psi_{2,1}\rangle$ to obtain the measurement result ${\left(p_{2}\right)}^{\prime }$ and compares ${\left(p_{2}\right)}^{\prime }$ with the published random number $p_{2}=12$. If ${\left(p_{2}\right)}^{\prime }=p_{2}=12$, Bob${}_{1}$ believes that all the information comes from Bob${}_{2}$ and Bob${}_{2}$ has received an own message. So, Bob${}_{1}$ will send the auxiliary state ${|k\rangle }_{T}$ in his own hand to Bob${}_{2}$ through the secure quantum channel at moment $t_{1,2}=9$.

**(V)** After Bob${}_{2}$ receives ${|k\rangle }_{T}$ at moment $t_{1,2}=9$, he treats ${|k\rangle }_{T}$ as the control bit and $|S_{2}\rangle =|14\rangle$ as the target bit. He performs controlled black box operation $C_{k}$ on these two quantum states. After performing the controlled black box operation, Bob${}_{2}$ next conducts the direct product operation on $|S_{2}\rangle =|14\rangle$ and ${|k\rangle }_{T}$. Then the whole quantum state system becomes $|\phi_{3}\rangle$.

(35) $$\begin{array}{cc} |\phi_{3}\rangle & =\left(I⊗I⊗C_{k}\right)(\frac{1}{\sqrt{17}}\sum_{k=0}^{16}\omega^{13k}|k\rangle_{H}{|k\rangle }_{T}\left|14\rangle \right)\\ \; & =\frac{1}{\sqrt{17}}\sum_{k=0}^{16}\omega^{13k}{|k\rangle }_{H}{|k\rangle }_{T}U^{k}|14\rangle \\ \; & =\frac{1}{\sqrt{17}}\sum_{k=0}^{16}\omega^{13k}{|k\rangle }_{H}{|k\rangle }_{T}\omega^{14k}|14\rangle \\ \; & =\frac{1}{\sqrt{17}}\sum_{k=0}^{16}\omega^{(13+14)k}{|k\rangle }_{H}{|k\rangle }_{T}|14\rangle . \end{array}$$

**(VI)** Each participant Bob${}_{i}$ and Bob${}_{i+1}$ repeat the above mutual authentication and operation process of Bob${}_{1}$ and Bob${}_{2}$. When Bob${}_{2}$ and Bob${}_{3}$ complete mutual authentication, Bob${}_{2}$ will send the auxiliary state ${|k\rangle }_{T}$ in his own hand to Bob${}_{3}$ through the secure quantum channel at moment $t_{2,3}=15$. Bob${}_{3}$ also performs a similar controlled black box operation first. Then, he performs the direct product operation on his quantum state $|S_{3}\rangle =|3\rangle$ and the whole quantum system, and so on, until the last participant Bob${}_{4}$ completes the direct product operation. At this time, the whole quantum system becomes $|\phi_{4}\rangle$.

(36) $$\begin{array}{cc} |\phi_{4}\rangle & =\frac{1}{\sqrt{17}}\sum_{k=0}^{16}\omega^{(13+14+3+6)k}{|k\rangle }_{H}{|k\rangle }_{T}|14\rangle |3\rangle |6\rangle \\ \; & =\frac{1}{\sqrt{17}}\sum_{k=0}^{16}\omega^{2k}{|k\rangle }_{H}{|k\rangle }_{T}|14\rangle |3\rangle |6\rangle . \end{array}$$

**(VII)** When Bob${}_{4}$ completes the direct product operation, Bob${}_{4}$ completes the identity authentication process with Bob${}_{1}$ in the same way. After completing the authentication operation, Bob${}_{4}$ retransmits the auxiliary state ${|k\rangle }_{T}$ back to Bob${}_{1}$ through a secure quantum channel. After Bob${}_{1}$ receives the auxiliary state ${|k\rangle }_{T}$ again, he performs a CNOT operation on the two particles in his hand, where ${|k\rangle }_{H}$ is control bit and ${|k\rangle }_{T}$ is target bit. At this time, the whole quantum system becomes $|\phi_{5}\rangle$.

(37) $$\begin{array}{cc} |\phi_{5}\rangle & =(CNOT(\frac{1}{\sqrt{17}}\sum_{k=0}^{16}\omega^{2k}|k\rangle_{H}|k\rangle_{T}))|14\rangle |3\rangle |6\rangle \\ \; & =\frac{1}{\sqrt{17}}\sum_{k=0}^{16}\omega^{2k}{|k\rangle }_{H}{|0\rangle }_{T}|14\rangle |3\rangle |6\rangle . \end{array}$$

**(VIII)** Bob${}_{1}$ uses computational basis to measure the quantum state ${|k\rangle }_{T}$ which has been handled by CNOT operation. If the measurement result is |0〉, Bob${}_{1}$ believes that his auxiliary particles have not been destroyed or replaced. Bob${}_{1}$ will continue to perform the following steps. Otherwise Bob${}_{1}$ has reason to believe that the auxiliary state is damaged or replaced during the transmission process, thus ending the entire agreement.

**(IX)** Bob${}_{1}$ applies IQFT on his first quantum state ${|k\rangle }_{H}$ and measures the output to obtain the final secret $S^{\prime }=2$.

(38) $$\begin{array}{cc} \; & \mathrm{IQFT}⊗I(\frac{1}{\sqrt{17}}\sum_{k=0}^{16}\omega^{2k}|k\rangle_{H}{|0\rangle }_{T})\\ = & (\frac{1}{\sqrt{17}}\sum_{k=0}^{16}\omega^{2k}\mathrm{IQFT}|k\rangle_{H}{)|0\rangle }_{T}\\ = & (\frac{1}{\sqrt{17}}\sum_{k=0}^{16}\omega^{2k}\left(\frac{1}{\sqrt{17}}\sum_{l=0}^{16}\omega^{-lk}\right)|l\rangle_{H}{)|0\rangle }_{T}\\ = & (\frac{1}{17}\sum_{k=0}^{16}\sum_{l=0}^{16}\omega^{(2-l)k}|l\rangle_{H}{)|0\rangle }_{T}\\ = & (\frac{1}{17}\sum_{k=0}^{16}|2\rangle_{H}+\frac{1}{17}\sum_{l=0,l\neq 2}^{16}\left(\sum_{k=0}^{16}\omega^{2-l)k}\right){|l\rangle }_{H}{)|0\rangle }_{T}\\ = & {(|2\rangle }_{H}+\frac{1}{17}\sum_{l=0,l\neq 2}^{16}{0|l\rangle }_{H}{)|0\rangle }_{T}\\ = & {|2\rangle }_{H}{|0\rangle }_{T}. \end{array}$$

**(X)** Bob${}_{1}$ calculates $H^{\prime }=h\left(2\right)$ according to hash function h() released by Alice and compares with public H=h(S). If $H^{\prime }=H$, $S^{\prime }$, the secret obtained by ${\mathrm{Bob}}_{1}$ is the real secret. If not, ${\mathrm{Bob}}_{1}$ has reason to believe that there is at least one dishonest participant, thus terminating the agreement.

## 8. Conclusions

In this article, using QFT, IQFT, mutually unbiased bases, and other relevant knowledge, we propose a quantum secret-sharing scheme that both sides of the communication can mutually verify the identity. Each participant holds his own share which will neither be disclosed nor transferred. Only at the secret-recovery stage, each participant will directly integrate his information into the whole quantum system, which avoids being stolen. Any participant has reason to recover the secret and only the reconstructor obtains the secret and is responsible for it. Since only *t* participants can recover the secret, the protocol is more flexible and practical. After our analysis, the protocol can resist intercept–resend attacks, entanglement–measurement attacks, collusion attacks, and forgery attacks, so it is safe enough.

## Figures and Tables

**Table 1:** Comparison of parameters among our protocol and previous protocols.

Protocols	Yang [3]	Song [7]	Sutradhar [8]	Mashhadi [9]	Our
(t,n)threshold	N	Y	Y	Y	Y
QFT	*n*	1	2	1	1
IQFT	-	1	2	*t*	1
measurement operation	*n*	1	2	*t*	2t+1
dimensional space	*d*	*d*	*d*	*d*	*d*
message particle	n−1	*t*	t+1	*t*	3t+1
hash function	2	2	2	2	2
intercept–resend	-	Y	Y	N	Y
entangle–measure	-	Y	Y	Y	Y
collusive attack	N	Y	N	N	Y
forgery attack	-	Y	Y	Y	Y
identity authentication	N	N	N	N	Y

## Data Availability

The relevant data in Section 7 is arbitrarily selected and calculated by us.
